# Sex-specific toxicity targets of aristolochic acids: nephrotoxicity in males, hepatotoxicity in females

**DOI:** 10.1007/s00204-025-04297-5

**Published:** 2026-01-21

**Authors:** Hong-Ching Kwok, Nikola M. Pavlović, Zongwei Cai, Wan Chan

**Affiliations:** 1 Department of Chemistry, The Hong Kong University of Science and Technology, Clear Water Bay, Kowloon, Hong Kong; 2 Faculty of Medicine, University of Niš, Bulevar Dr Zorana Đinđića 81, Niš, 18000 Serbia; 3 Eastern Institute of Technology Ningbo, Ningbo, 315200 Zhejiang China

**Keywords:** Aristolochic acids, Sex difference, DNA damage, Nephrotoxicity, Hepatotoxicity

## Abstract

Aristolochic acids (AAs), derived from *Aristolochia* herbs, are well-known nephrotoxins, and emerging evidence suggests their potential role in the development of liver cancers. However, the specific organs most affected by AAs, particularly in relation to liver cancer, remain unclear. Considering the known sex differences in enzyme activities, we hypothesized that variations in AA metabolism may contribute to the kidney and liver toxicity associated with these compounds. Our analysis of DNA adducts in the kidneys and livers of mice treated with aristolochic acid I (AA-I) revealed that male mice exhibited over 2.5 times higher levels of DNA adducts in their kidney DNA compared to female mice. Conversely, female mice showed 1.5 times higher adduct levels in their liver DNA than their male counterparts. These findings indicate that AA exposure presents a sex-specific disease risk, with males being at greater risk for kidney disease and females for liver disease. Additionally, we observed similar concentration patterns of the metabolite aristolactam I (AL-I) and the activity of NQO1 enzymes in the respective organs. Further in vitro studies, involving the incubation of AA-I with liver and kidney homogenates, demonstrated significant differences in AL-I concentrations, mirroring the trends observed in the AA-DNA adduct and AL-I analyses of AA-I-exposed mice. Collectively, these results underscore the importance of sex differences in the enzymatic activity responsible for the metabolic activation of AAs, which is critical for understanding the differential nephrotoxic and hepatotoxic effects associated with these compounds.

**Supplementary Information:**

The online version contains supplementary material available at 10.1007/s00204-025-04297-5.

## Introduction

**Fig. 1:**
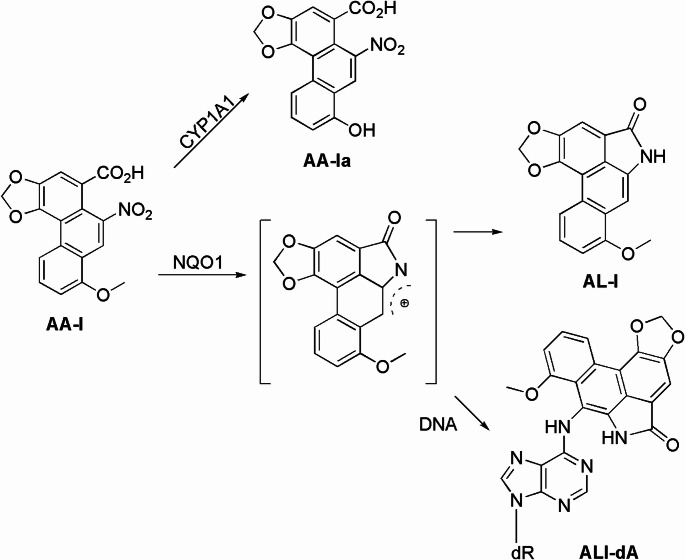
Metabolic activation, deactivation, and DNA adduct formation of aristolochic acid I

Aristolochic acids (AAs; Fig. 1) are naturally occurring nitrophenanthrene carboxylic acids produced by plants in the *Aristolochia* and *Asarum* genera, many of which have been historically utilized in herbal medicine (Ang et al. 2021; Jou et al. 2003; Hashimoto et al. 1999). Since the early 1990s, however, reports of AA poisoning associated with the use of AA-laden herbal remedies have emerged from countries such as Australia, China, Hong Kong, Japan, Taiwan, and the U.K. (Debelle et al. 2008; Chan et al. 2006). These reports indicate that the consumption of herbal products containing AAs significantly contributes to conditions like kidney fibrosis and upper tract urothelial cancer (UTUC), collectively referred to as Chinese herb nephropathy, which was later renamed aristolochic acid nephropathy (AAN) to accurately reflect its causative agent *(*Ang et al. 2021; Debelle et al. 2008; Chan et al. 2006; Chen et al. 2012). Controversially, there are suggestions that AA-containing herbs may also be linked to liver cancers in Taiwan and across Asia (Li et al. 2024; Ng et al. 2017; Poon et al. 2013; Shinde et al. 2017; Lu et al. 2020; Chen et al. 2020; Fang et al. 2022; Xian et al. 2021).

In response to these alarming incidents, many countries have prohibited the sale and use of most AA-containing herbs in medical treatments (Debelle et al. 2008; Cheung et al. 2006). The U.S. FDA has mandated the cessation of AAs in the production of herbal remedies (Zhang et al. 2019; USFDA 2001), and the International Agency for Research on Cancer (IARC) of the World Health Organization (WHO) has classified AAs and AA-containing herbs as Group I carcinogens (Debelle et al. 2008; Anttila et al. 2002). Despite these measures, AA-containing herbs remain readily available from various sources, and misuse continues to pose a significant risk (Debelle et al. 2008; Gökmen et al. 2013). It is estimated that over 100 million individuals, particularly in Asia, are at risk of AA exposure due to the widespread use of herbal medicines (Grollman 2013; Hu et al. 2004; Vanherweghem et al. 1993; Dickman et al. 2023; Hsieh et al. 2008).

Recent studies have shown that AAs are environmental pollutants in some European countries, such as Romania, Bulgaria, and Serbia and parts of China (Jadot et al. 2017). In particular, environmental AAs-exposure through contaminated food is causing the Balkan endemic nephropathy (BEN), a chronic kidney disease that shares similar clinical characteristic as the AAN, in the Balkan Peninsula (Jadot et al. 2017). It was estimated that around 100,000 individual are at risk of AA-associated UTUC and kidney failure (Jadot et al. 2017).

Emerging evidence suggests that AAs exert stronger nephrotoxicity in male mice compared to female mice (Shi et al. 2016; Li et al. 2023; Wang et al. 2025), aligning with observations from the United States Renal Data System, which indicate that the adjusted incidence of total end-stage renal disease (ESRD) in men is approximately 60% higher than that in women (USRDS 2023). It has been proposed that estradiol, the female sex hormone, reduces the activity of organic anion transporters, thereby decreasing the accumulation of AAs and their toxic metabolites in renal tubules, which alleviates nephrotoxicity in female mice (Wang et al. 2025). However, the exact mechanisms underlying these observed differences in nephrotoxicity remain incompletely understood, and potential differences in carcinogenicity and hepatotoxicity between male and female mice have yet to be explored.

The goal of this study is to investigate the poorly understood sex differences in AAN using chemical methods. Given that DNA damage is believed to be associated with both the nephrotoxicity and carcinogenicity of AAs (Cosyns 2003; Arlt et al. 2002; Zhang et al. 2022), we initiated our study by quantifying exposure-specific AA-DNA adduct levels in DNA isolated from the kidneys and livers of both male and female mice exposed to aristolochic acid I (AA-I) using a liquid chromatography-tandem mass spectrometry (LC-MS/MS) coupled with a stable isotope dilution method. We then quantified the levels of AA-I and its major metabolites, aristolactam I (AL-I; Fig. 1) and aristolochic acid Ia (AA-Ia), in the kidneys and livers of AA-I-exposed mice. Our findings reveal for the first time that AA-I exerts greater nephrotoxicity in male mice while exhibiting higher hepatic toxicity in females likely due to differences in the activities of enzymes involved in the metabolic activation and deactivation of AAs. This pattern of sexual dimorphism in drug-metabolizing enzymes has also been observed with various other toxicants and drugs (Ueno et al. 2012; Tamargo et al. 2017; Los et al. 1996). In addition to elucidating the molecular mechanisms underlying the observed nephrotoxicity in male mice, our results may help clarify the ongoing debate regarding the association between AA exposure and liver cancer in humans.

## Materials and methods

### Materials

 All chemicals and reagents utilized in this study were of the highest available purity and were employed without further purification unless specifically mentioned. AA-I was sourced from Acros Organics (Morris Plains, NJ). The compounds AL-I, AA-Ia, ALI-dA, and the ^15^N_5_-ALI-dA internal standard were obtained from previous studies (Liu et al. 2019; Au et al. 2020). The chemicals required for analyzing DNA adducts and AA metabolites—including alkaline phosphatase, DNase I, nuclease P1, snake venom phosphodiesterase, benz[*cd*]indol-2(1*H*)-one, and 17β-estradiol—were acquired from Sigma (St. Louis, MO). S9 fractions were prepared following a procedure published previously with some modifications (Tang et al. 2012), as detailed in the SI Appendix. Milli-Q water was generated through a purification system using a PALL Cascada I water purification system (Port Washington, NY).

### Instrumentation

 Quantitative analysis of ALI-dA was conducted using a Waters Acquity UPLC system linked to a TQ-XS triple quadrupole LC–MS/MS system (Waters Corp., Milford, MA), as described previously (Zhang et al. 2022, 2023; Liu et al. 2019; Guo et al. 2024; Au et al. 2023; Chan and Ham 2021; Kwok et al. 2025). The metabolites of AA-I in tissue samples and the S9 reaction mixture were quantified using a Waters Acquity UPLC paired with an AB Sciex API 4000 QTRAP, as stated in previous studies (Zhang et al. 2022; Ham et al. 2024). Both analytical setups utilized positive electrospray ionization and operated in multiple reaction monitoring (MRM) mode, with the mass spectrometry (MS) parameters provided in the SI Appendix, Table S1.

### HPLC methods

Two HPLC methods utilizing the same column (Luna C18; 3-µm particle size; 2 × 100 mm; Phenomenex Inc.; Torrance, CA) were employed for the analysis of ALI-dA (method 1) and AA/metabolites (method 2), as detailed below.

*HPLC Method 1.* The column was equilibrated with 98% solvent A (0.1% acetic acid in water) and 2% solvent B (acetonitrile) at a flow rate of 0.35 mL/min at 40 °C. The solvent gradient was programmed as follows: a linear increase from the initial solvent to 30% (v/v) B in 1 min; increasing from 30 to 70% (v/v) B in 3 min; raising to 100% B in 1 min, holding for 4 min; decreasing to 2% B in 0.1 min; and re-equilibrating at initial conditions for 2 min.

*HPLC Method 2.* The column was equilibrated with 99% solvent A (0.2% acetic acid and 0.01 M ammonium acetate in water) and 1% solvent B (acetonitrile) at a flow rate of 0.4 mL/min. The solvent gradient was programmed as follows: a linear increase from the starting solvent to 99% (v/v) B in 6 min; holding for 2 min; decreasing to 1% B in 0.1 min; and re-equilibrating at initial conditions for 2 min.

### Administration of AA-I and/or Estrogen to C57BL/6 mice

 Animal experiments were carried out in accordance with the protocols approved by the Animal Ethics Committee at HKUST (AEP-2023-0041). Male and female C57BL/6 mice (8–9 weeks) were obtained from the HKUST Laboratory Animal Facility. The mice were kept in a controlled environment with a 12-h light/dark cycle, and were provided food and water *ad libitum*. After a week of acclimatization, the mice were randomly assigned to receive varying doses of AA-I (*n* = 5; 5, 10, or 20 mg/kg) in a 0.1 M NaHCO_3_ solution. The control group (*n* = 5) was administered the dosing vehicle. All mice were euthanized by decapitation 6-h after dosing, and their kidneys and livers were harvested and stored at – 80 °C for further analysis. Additionally, to evaluate time-dependent effects, the mice were divided into nine groups (*n* = 5) and given a single oral dose of AA-I at 10 mg/kg in a 0.1 M NaHCO_3_ solution. These mice were sacrificed at intervals of 0, 2, 4, 6, 8, 16, 24, 48, and 72 h post-dosing, and their organs were collected for further investigation. For estrogen-treated groups, male and female mice received different doses (0, 0.5, or 2 mg/kg/day) of 17β-estradiol, dissolved in pure corn oil, by daily subcutaneous injections for one week prior to the oral administration of AA-I (10 mg/kg). Vehicle-treated mice received the same volume of corn oil and were also given 10 mg/kg of AA-I orally. All mice were sacrificed 6-h after AA treatment, and their organs were collected for further analysis.

### DNA isolation and digestion

 DNA was extracted from the kidneys and livers of mice exposed to AA using the Omega Bio-tek DNA extraction kit (Norcross, GA), following the manufacturer’s guidelines. The extracted DNA (approximately 15 µg dissolved in 100 µL of water) was mixed with 15 µL of an internal standard solution containing 0.1 nM ^15^N_5_-ALI-dA before undergoing enzymatic digestion with nuclease P1, DNase I, alkaline phosphatase, and snake venom phosphodiesterase, as described in previous studies (Zhang et al. 2022; Guo et al. 2024; Chan and Ham 2021; Au et al. 2020). The resulting DNA hydrolysates were then centrifuged at 13,800 *rcf* at 4 °C for 10 min before analysis using the LC − MS/MS method and HPLC Method 1.

### Organ preparation for AA-I metabolites analysis

 To assess the levels of AA-I metabolites in the kidneys and livers of mice, approximately 50 mg of each organ was weighed and rinsed with ice-cold phosphate-buffered saline (PBS) before being homogenized in 0.5 mL of PBS, as previously described (Zhang et al. 2022; Ham et al. 2024). In brief, 200 µL of the resulting homogenate was taken and mixed with four times its volume of ice-cold acetone to precipitate proteins. The protein concentration in the sample was measured using Merck BCA protein assay kits (St. Louis, MO) according to the manufacturer’s guidelines. The supernatants were then evaporated using a nitrogen stream, and the resulting residues were dissolved in 50 µL of 80% methanol for subsequent LC − MS/MS analysis with HPLC Method 2.

### NQO1 enzyme activity

 Male and female mice without AA-I treatment, and with varying doses of 17β-estradiol were utilized to assess NQO1 enzyme activity. Fresh tissue samples were collected and analyzed for NQO1 activity using an NQO1 activity assay kit (Abcam, Cambridge, UK) following the manufacturer’s instructions. The resulting NQO1 activity levels were then normalized according to the protein concentration measured using a Merck BCA protein assay.

### Incubation of AA-I with mice liver and kidney S9 fraction

 AA-I (200 µM) was introduced into a solution containing 3.3 mM MgCl_2_ and 8 mg/mL S9 fraction in 100 mM potassium phosphate at pH 7.4, along with an NADPH generating system (1.3 mM NADP, 3.3 mM glucose 6-phosphate, and 0.4 units/mL of glucose 6-phosphate dehydrogenase), and incubated at 37 °C. Reaction mixtures (100 µL) were collected at time points of 0, 0.5, 1, 1.5, 2, 3, and 4 h. The samples underwent three extractions with 300 µL of ethyl acetate. The extracts were then evaporated to dryness under a nitrogen stream, and the resulting residues were reconstituted in 100 µL of 80% methanol containing 60 nM of the internal standard benz[*cd*]indol-2(1*H*)-one. The reaction mixtures were subsequently analyzed using LC-MS/MS with HPLC Method 2. Control experiments were performed in the same manner, except that the S9 fraction was omitted.

### Statistical analysis

All data were analyzed using GraphPad software and are presented as the mean ± standard deviation (SD) from five independent experimental trials (*n* = 3 for the studies of incubation of AA-I with liver and kidney S9 fraction). Comparisons between the control and experimental groups were performed using a two-tailed unpaired Student’s *t*-test with a 95% confidence interval. The significance levels were categorized as follows: *ns p* > 0.05, * *p* < 0.05, ** *p* < 0.01, *** *p* < 0.001, **** *p* < 0.0001.

## Results

**AA-DNA adduct levels in kidneys of AA-I-exposed male and female mice**

 The initial analysis focused on quantifying the level of 7-(deoxyadenosin-*N*^6^-yl)-aristolactam I (ALI-dA), the most mutagenic and predominant DNA adduct of AA-I (Cosyns 2003; Arlt et al. 2002), in the kidney DNA of AA-I-exposed mice. The kidney was selected as the primary organ of interest due to its well-documented role as a target for tumorigenesis associated with AAs and its recognized nephrotoxicity (Chen et al. 2012, 2020; Fang et al. 2022; Xian et al. 2021; Liu et al. 2019; Grollman et al. 2007). Following hydrolysis using our previously developed enzyme digestion method, we rigorously quantified the ALI-dA adduct in the isolated kidney DNA using a stable isotope-dilution LC-MS/MS method (Guo et al. 2024; Zhang et al. 2023; Au et al. 2023; Chan and Ham 2021; Kwok et al. 2025).

**Fig. 2:**
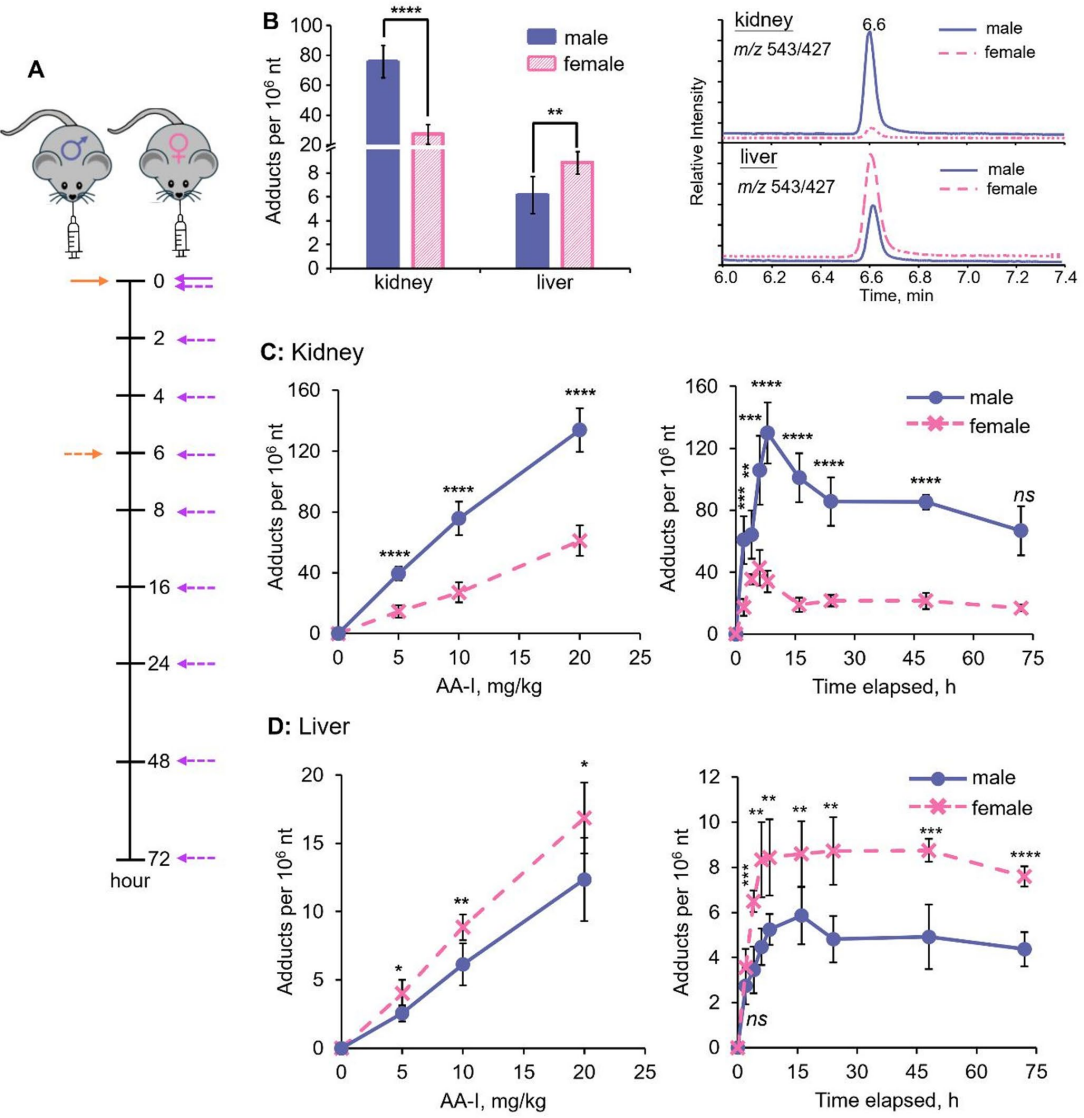
Sex differences in DNA adduct formation of aristolochic acid I in the kidneys and livers of exposed male and female mice. **a** Schematic illustration of animal experiment. Male and female mice were orally administered with a single dose of AA-I (for time-course study) or with different doses of AA-I (for dose-dependent study). While solid arrows indicate the time point that mice administered with AA-I, orange and purple dotted arrow indicate the time points that mice were sacrificed for dose-dependent and time-course study, respectively. **b** DNA adduct levels in the kidneys and livers of male (*n* = 5) and female (*n* = 5) mice treated with a single oral dose of 10 mg/kg body weight AA-I, along with reconstructed chromatograms from LC-MS/MS analysis of ALI-dA adduct in the kidney and liver DNA of AA-I-treated male and female mice. DNA adduct levels in the **c** kidneys and **d** liver of male (*n* = 5) and female (*n* = 5) mice treated with varying amounts of AA-I (left), and time profiles of adduct levels (right) in male (*n* = 5) and female (*n* = 5) mice treated with a single oral gavage of 10 mg/kg body weight AA-I. The data represent mean ± SD from five independent experiments. A two-tailed unpaired Student’s *t*-test at a 95% confidence interval was conducted to compare AA-DNA adduct levels in the kidneys and livers of male and female mice treated with the same dose of AA-I at the same time point. *ns p* > 0.05; *, *p* < 0.05; **, *p* < 0.01; ***, *p* < 0.001; ****, *p* < 0.0001

The analysis revealed a significant difference in ALI-dA adduct levels between the exposed mice (*p* < 0.0001; Fig. 2), with levels approximately 2.8 times higher in the kidneys of male mice compared to female mice. Specifically, ALI-dA was detected at 75.7 ± 11 adducts per 10^6^ nucleotides in male mice and 27.0 ± 6.5 adducts per 10^6^ nucleotides in female mice that received a single oral dose of AA-I at 10 mg/kg. A linear dose-dependent relationship was observed (Fig. 2), with a similar trend of higher adduct levels in male mice fed increasing amounts of AA-I.

Further analysis of DNA adducts in kidney samples collected at different time points after AA-I exposure revealed a significantly higher adduct formation rate (15.2 ± 2.3 adducts per 10^6^ nt/h vs. 7.33 ± 1.6 adducts per 10^6^ nt/h; Fig. 2C) in the kidneys of male mice compared to female mice, along with a significantly higher persistence of the adduct in male mice.

**Fig. 3:**
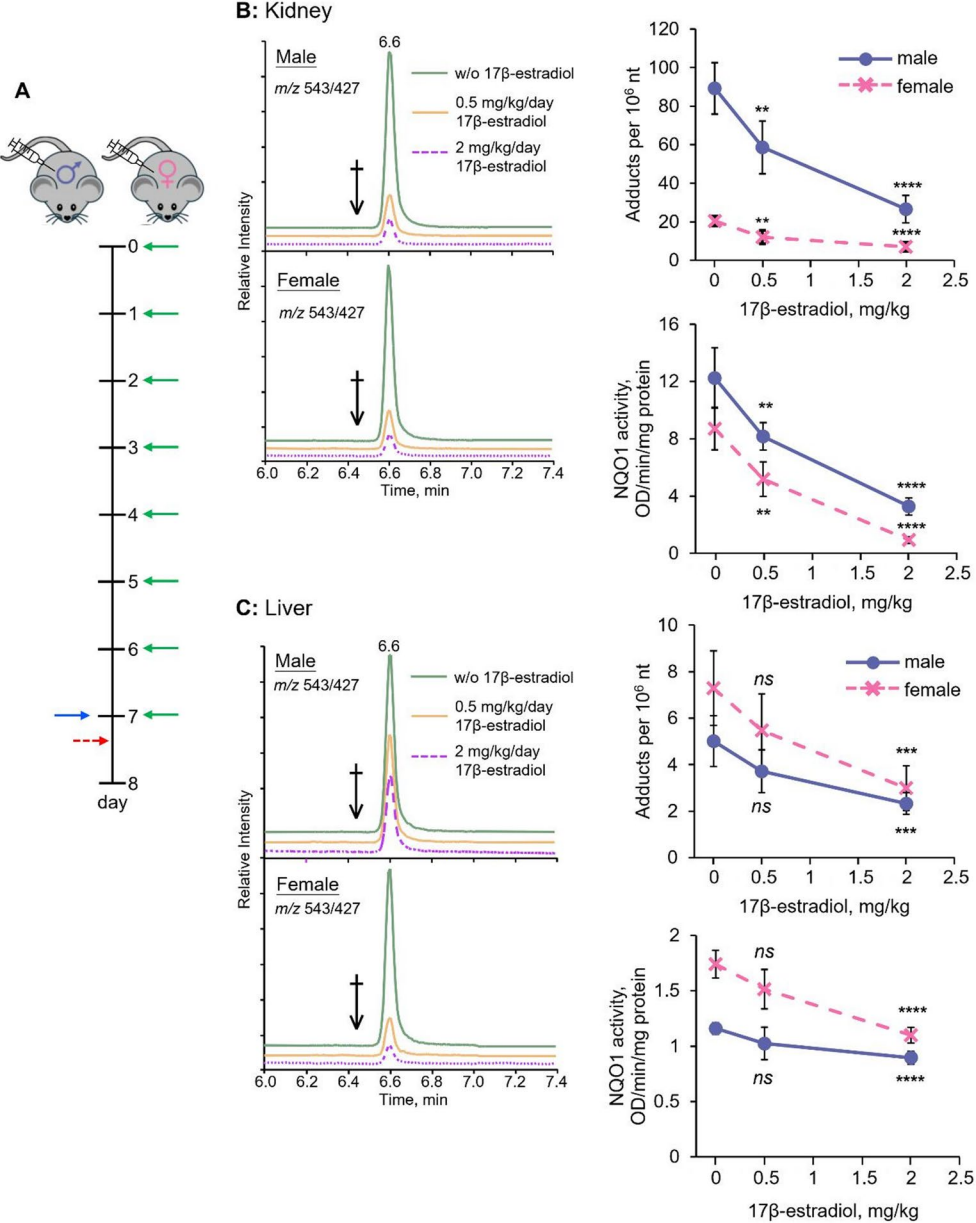
Sex hormones affect activity of activation enzymes, thus DNA adduct formation of aristolochic acid I, in kidneys and livers of exposed male and female mice. **a** Schematic illustration of animal experiment. Male and female mice were subcutaneously injected daily with different doses of 17β-estradiol for one week before received single oral gavage of AA-I, and sacrificed 6 h after AA treatment. Green and blue solid arrows indicate the time points that mice received subcutaneous injection of 17β-estradiol and oral gavage of AA-I, respectively. Red dotted arrow indicates the time point that the mice were sacrificed. Reconstructed chromatograms from LC-MS/MS analysis of AA-DNA adduct, AA-DNA adduct levels and NQO1 activity in **b** kidneys and **c** livers of male (*n* = 5) and female (*n* = 5) mice pretreated with different doses of 17β-estradiol for one week and before receiving a single oral administration with 10 mg/kg AA-I. The data represent mean ± SD for five independent experiments. 2-tailed unpaired Student’s *t*-test at 95% confidence interval was conducted to compare the level AA-DNA adduct in kidneys and livers of male and female mice between control and 17β-estradiol treatment groups. *ns p* > 0.05; **, *p* < 0.01; ***, *p* < 0.001; ****, *p* < 0.0001

Next, we investigated the potential role of sex hormones in the observed sex-specific toxicity of AA-I. To this end, we administered 17β-estradiol subcutaneously to mice for one week prior to AA exposure. Compared to control mice that received the dosing vehicle, results showed a general dose-dependent decrease in ALI-dA adduct levels in the kidneys of both male and female mice treated with estrogen (Fig. 3). These findings are in excellent agreement with previous proposal of a crucial role for sex hormones in the observed nephrotoxicity of AA, leading to the preferential formation of DNA adducts in the kidneys of male mice compared to female mice.

### AA-DNA adduct levels in liver of AA-I-exposed male and female mice

 Analysis of liver DNA revealed that adduct levels were generally ten times lower compared to kidney DNA in both male and female mice (Fig. 2). Notably, the data showed an intriguing phenomenon: ALI-dA levels were 1.5 times higher in females than in males, with adduct levels detected at 8.9 ± 0.9 adducts per 10^6^ nucleotides in females and 6.1 ± 1.5 adducts per 10^6^ nucleotides in males who received a single oral dose of AA-I at 10 mg/kg.

Similar to that observed in the kidney DNA samples, a dose-dependent suppressive effect of estrogen on ALI-dA adduct formation was observed in estrogen-treated mice (Fig. 3). The data also indicated a significantly higher adduct formation rate (1.40 ± 0.2 adducts per 10^6^ nt/h vs. 0.61 ± 0.07 adducts per 10^6^ nt/h; Fig. 2D) in the livers of female mice compared to males, along with a significantly higher persistence of the adduct in female mice. These results provide the first direct evidence that AA-I is more hepatotoxic in females than in males.

### Concentrations of AA-Ia and AL-I in kidney and liver of AA-I-exposed male and female mice

**Fig. 4:**
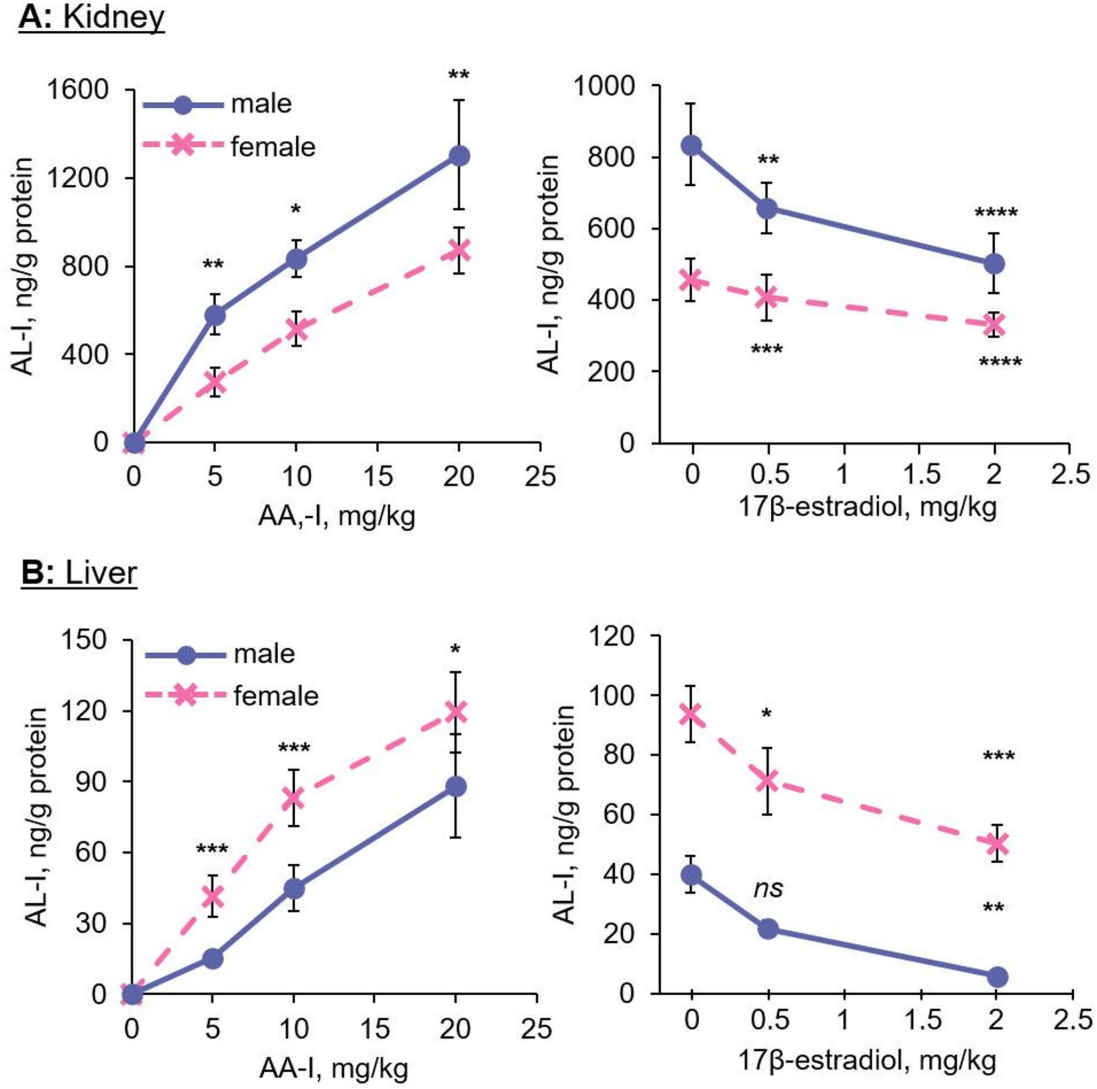
Sex hormones affect the metabolic activation of aristolochic acid I, thus the concentrations of aristolactam I in kidneys and livers of exposed male and female mice. Aristolactam I levels in **a** kidneys and **b** livers of male (*n* = 5) and female (*n* = 5) mice treated with a single oral gavage containing different doses of AA-I, and pretreated with different doses of 17β-estradiol for one week before receiving a single oral administration with 10 mg/kg AA-I. The data represent mean ± SD for five independent experiments. 2-tailed unpaired Student’s *t*-test at 95% confidence interval was conducted to compare the level of aristolactam I in kidney and liver of male and female mice treated with same doses of AA-I or between control and 17β-estradiol treatment groups. *ns p* > 0.05; *, *p* < 0.05; **, *p* < 0.01; ***, *p* < 0.001; ****, *p* < 0.0001

 Using an LC-MS/MS method, we determined the concentrations of AA-Ia and AL-I in the kidneys and livers of AA-I-exposed mice. The results revealed a similar concentration pattern for AL-I and the ALI-dA adducts in both kidney and liver samples (Fig. 4). Specifically, AL-I concentrations were higher in the kidneys of male mice compared to female mice, while liver concentrations were greater in female mice than in males. Notably, administration of estrogen decreased AL-I concentrations in both organs in a concentration-dependent manner.

**Fig. 5:**
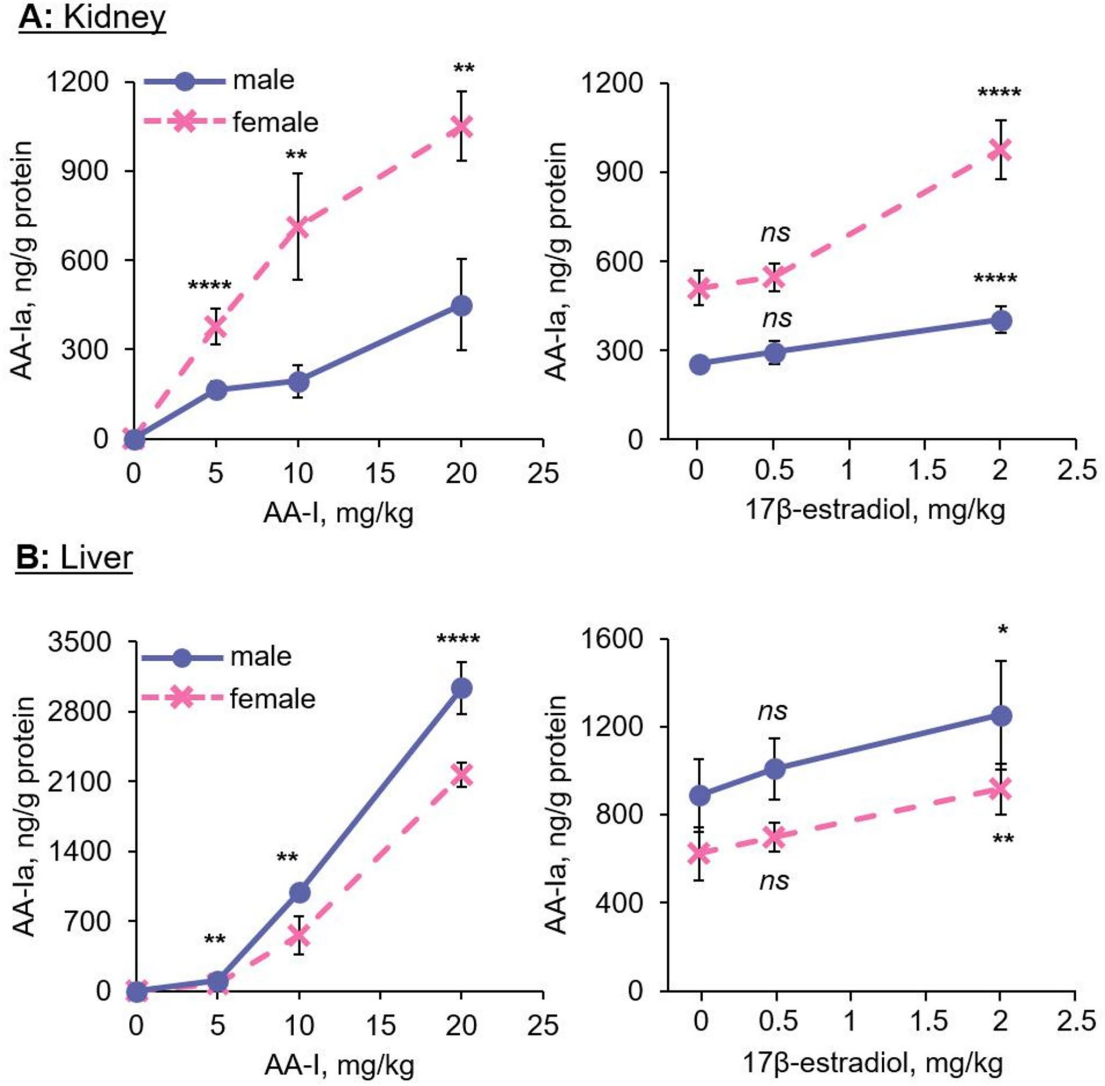
Sex hormones affects the metabolic deactivation of aristolochic acid I, thus the concentrations of aristolochic acid Ia in kidneys and livers of exposed male and female mice. Aristolochic acid Ia levels in **a** kidneys and **b** livers of male (*n* = 5) and female (*n* = 5) mice treated with a single oral gavage containing different doses of AA-I, and pretreated with different doses of 17β-estradiol for one week before receiving a single oral administration with 10 mg/kg AA-I. The data represent mean ± SD for five independent experiments. 2-tailed unpaired Student’s *t*-test at 95% confidence interval was conducted to compare the level of aristolochic acid Ia in kidney and liver of male and female mice treated with same doses of AA-I or between control and 17β-estradiol treatment groups. *ns p* > 0.05; *, *p* < 0.05; **, *p* < 0.01; ***, *p* < 0.001; ****, *p* < 0.0001

In contrast, higher concentrations of AA-Ia were detected in the kidneys of female mice compared to males (Fig. 5), while liver concentrations of AA-Ia were higher in male mice than in females. Furthermore, in both male and female mice administered estrogen, we observed an estrogen concentration-dependent increase in AA-Ia levels in both the kidneys and livers. These findings align with prior observations that estrogen enhances the activity of CYP1A1, a key enzyme involved in the metabolic deactivation of AA-I, leading to increased production of AA-Ia (Penaloza et al. 2014; Priestap et al. 2021).

### NQO1 activity in kidney and liver male and female mice

 In a separate experiment, we measured the activity of NQO1, a key enzyme involved in the activation of AAs (Fig. 1) (Priestap et al. 2021; Stiborová et al. 2017; Stiborová et al. 2013), in the kidneys and livers of both male and female mice that were not treated with AAs. The results indicated a consistent trend of higher NQO1 activity in the kidneys compared to the livers (Fig. 3). Specifically, NQO1 activity was significantly higher in the kidneys of male mice than in those of female mice, while liver activity was greater in female mice compared to their male counterparts. These findings correspond with the observed DNA adduct levels in the kidneys and livers of AA-I-exposed mice, showing higher DNA adduct levels in the kidney DNA of males and in the liver DNA of females (Fig. 2). Additionally, administration of estrogen was found to reduce NQO1 activity in a concentration-dependent manner (Fig. 3).

### Incubation of AA-I with mouse liver and kidney S9

 The possibility of sex-specific differences in the activities of AA-metabolizing enzymes, which may contribute to the observed sex differences in AA toxicity, was further validated by incubating AA-I with S9 fractions prepared from the kidneys and livers of male and female mice. To this end, we incubated AA-I with S9 derived from both kidney and liver tissues of male and female mice, and the levels of AL-I and AA-Ia resulting from metabolic activation and deactivation were determined using LC-MS/MS.

The analysis revealed higher levels of AL-I produced in the AA-I-incubated S9 fraction prepared from the kidneys of male mice compared to female mice (Figure S1). In contrast, higher levels of AL-I were detected in the livers of female mice than in those of male mice, with an opposite trend observed for AA-Ia (Figure S2). These results, which are consistent with the levels of AA-Ia and AL-I found in the kidneys and livers of AA-I-exposed mice described above, confirm the existence of sex-specific differences in the activity of key AA-metabolizing enzymes involved in the metabolic activation of AAs. This variation may affect DNA adduct formation and potentially enhance their mutagenicity and carcinogenicity. Furthermore, it was demonstrated that the increased levels of AA-Ia (Figure S2) and the diminished levels of AL-I (Figure S1) were generated from S9 derived from both the renal and hepatic tissues of estrogen-treated male and female mice.

## Discussion

The growing recognition of sex differences in the nephrotoxicity of AAs has prompted efforts to quantify marker molecules such as blood creatinine, urea nitrogen, and proteins, aiming to elucidate the mechanisms underlying these biological differences (Shi et al. 2016; Li et al. 2023; Wang et al. 2025). We have applied highly accurate and sensitive LC-MS/MS techniques to quantify a major DNA adduct, ALI-dA, and two primary metabolites of AA-I, AL-I and AA-Ia, in the kidneys and livers of male and female mice exposed to AA-I, which is the most abundant and carcinogenic AA in *Aristolochia* herbs (Hashimoto et al. 1999; Debelle et al. 2008).

While previous studies of sex specific difference in AA toxicity to kidney targeted marker proteins of kidney fibrosis (Shi et al. 2016; Li et al. 2023), the results of our chemical analyses revealed significant quantitative features of ALI-dA, AL-I, and AA-Ia in the kidneys and livers of AA-I exposed male and female mice. The analysis showed generally higher levels of ALI-dA in the kidneys compared to the livers. Notably, we detected over 2.5 times higher concentrations of ALI-dA in kidney DNA of AA-I-exposed male mice compared to female mice, indicating higher nephrotoxicity and genotoxicity of AA-I in male mice than female mice. Because DNA damage is one of the key steps in chemical carcinogenesis, these findings are in good agreement with previous observations that the kidneys are key target organs in AA-mediated carcinogenesis, with cases of AA-associated kidney diseases reported globally (Debelle et al. 2008; Chen et al. 2012, 2020; Lu et al. 2020; Fang et al. 2022; Liu et al. 2019; Grollman et al. 2007).

Sex difference in hepatotoxicity of AAs has not been reported on the literature, in part due to the historical belief that liver is one of the non-target organs for AAs-mediated carcinogenesis (Fernando et al. 1993; Shibutani et al. 2007; Kohara et al. 2002; Chen et al. 2006). Measurement of ALI-dA in liver DNA of AA-I-exposed mice revealed higher levels of ALI-dA in female mice compared to male mice. These results point to greater hepatotoxicity and genotoxicity of AA-I in the livers of female mice, which is consistent with the recent observation of higher frequency of AA-specific mutation signatures observed in the livers of hepatocellular carcinoma (HCC) female patients compared to their male counterparts (Ng et al. 2017). Here, it is worth-mentioning that despite epidemiological studies suggesting a potential causative role of AAs in hepatocellular carcinoma (HCC) in humans (Ng et al. 2017; Poon et al. 2013; Letouzé et al. 2017; Lu et al. 2020), this role remains debated, particularly because tumors were not observed in the mice used in the experiments (Chen et al. 2020; Fang et al. 2022; Xian et al. 2021). The insufficient recognition of sex differences in AA toxicity may contribute to the argument that AAs do not cause liver cancer, especially considering that only male, but no female mice were used in these studies (Chen et al. 2020; Fang et al. 2022; Xian et al. 2021), while our findings suggest that the livers of female mice are more susceptible to AA-induced DNA damage.

Studies of DNA isolated from estrogen-treated mice revealed an estrogen-dependent reduction of ALI-dA adducts in the kidneys and livers of both male and female mice exposed to AA-I (Fig. 3). These results underscore the significant role of sex hormones in the activity of AA activation enzymes and, consequently, the toxicity of AAs.

While AA-I was not detected in the kidneys and livers of either male or female mice, the analysis of AL-I and AA-Ia in these organs revealed positive and negative correlations with ALI-dA adduct levels, respectively. Specifically, higher concentrations of AL-I were detected in the kidneys of male mice compared to female mice, whereas AL-I levels were higher in the livers of female mice than in males. Additionally, estrogen decreased AL-I concentrations in the kidneys and livers of both male and female mice in a concentration-dependent manner. Furthermore, an inverse correlation was observed between the concentrations of ALI-dA and AA-Ia, which is a major metabolite in the metabolic deactivation of AA (Priestap et al. 2012). Notably, estrogen treatment was previously showed to increase the activity of AA-deactivating enzymes, e.g. CYP1A1 (Penaloza et al. 2014; Priestap et al. 2012), thereby elevating concentrations of AA-Ia in the tested organs.

This crucial role of sex hormones in the observed sex differences in AAs toxicity is further supported by our measurements of NQO1 activity, a key enzyme known to be involved in the activation of AAs that leads to the production of AL-I and ALI-dA (Fig. 1) (Priestap et al. 2012; Stiborová et al. 2017; Stiborová et al. 2013), in mice not exposed to AAs. Specifically, we observed: (1) higher NQO1 activity in the kidneys of male mice compared to female mice; (2) higher enzyme activity in the livers of female mice compared to male mice; and (3) estrogen-induced dose-dependent reductions in NQO1 activity in the livers and kidneys of both female and male mice. These results align remarkably with the above-mentioned findings on quantifying ALI-dA and AL-I in the kidneys and livers of AA-I-exposed, estrogen-treated mice.

The important role of sex hormones in influencing the activities of enzymes in AA metabolism, thus the observed sex difference in AA toxicity, was further validated by incubating AA-I with S9 prepared from mice liver and kidney. Analysis of AL-I and AA-Ia in the incubated solution showed excellent agreement with the observation in the kidney and liver of AA-I exposed mice, in which higher levels of AL-I was detected in S9 prepared from male kidney than that of female, whereas higher levels of AL-I was detected in S9 prepared from female liver than that of male and that elevated levels of AA-Ia and decreased levels of AL-I (Figures S1 and S2) were produced from S9 prepared from both kidneys and livers of estrogen-treated male and female mice.

## Conclusion

The potential causative role of AAs in hepatocellular carcinoma remains controversial. Using male and female mice as the animal models, this study reveals for the first time that AAs exert sex-specific differences in toxicity, with higher nephrotoxicity observed in males and higher hepatotoxicity in females. These effects are mediated by differences in the activity of enzymes involved in the metabolic activation and deactivation of AAs, leading to DNA adduct formation in male and female mice. This finding suggests that some of the discrepancies in previous epidemiological studies regarding the hepatotoxicity of AAs in animal models may, in part, be attributed to the use of laboratory rodents of different sexes.

## Supplementary Information

Below is the link to the electronic supplementary material.

Supplementary Material 1

## Data Availability

All data are available in the main text or the supplementary materials.
