# Supplementary material for: Sex-specific toxicity targets of aristolochic acids: nephrotoxicity in males, hepatotoxicity in females
Source: Arch Toxicol. 2026 Jan 21;100(4):1575–87. doi: 10.1007/s00204-025-04297-5 (PMC13043579; doi:10.1007/s00204-025-04297-5)
Supplement: Supplementary file 1 — Supplementary Material 1 [file 204_2025_4297_MOESM1_ESM.docx]

**Supplementary Information**

**Sex-Specific Toxicity Targets of Aristolochic Acids: Nephrotoxicity in Males, Hepatotoxicity in Females**

*Hong-Ching Kwok ^a^, Nikola M. Pavlović ^b^, Zongwei Cai ^c^, and Wan Chan ^a,^**

^a^ Department of Chemistry, The Hong Kong University of Science and Technology, Clear Water Bay, Kowloon, Hong Kong

^b^ Medical Faculty, University of Niš, Bulevar Dr Zorana Đinđića 81, Niš 18000, Serbia

^c^ Eastern Institute of Technology Ningbo, Ningbo, Zhejiang 315200, China

* Corresponding authors: [chanwan@ust.hk](mailto:chanwan@ust.hk)

**Materials and Methods**

**Mice Liver and Kidney S9 Fraction Preparation.** Liver and kidney S9 fractions of male and female mice without any treatment or with administration of estrogen for one week were prepared following a procedure published previously with some modifications (Tang et al. 2012). In brief, three freshly harvested mice livers and kidneys were washed and then perfused with ice-cold saline, weighed, and minced. Minced livers and kidneys were homogenized using a Dounce homogenizer with ice-cold homogenization buffer (50 mM potassium phosphate, 250 mM sucrose, 1 mM EDTA, pH 7.4) (1g tissue/3 mL) and centrifuged at 9,000 *rcf* for 30 min at 4 °C. The supernatant was collected and stored at −80 °C until use. The protein concentration of the S9 fraction was determined by the Merck BCA protein assay kits (St. Louis, MO).

**
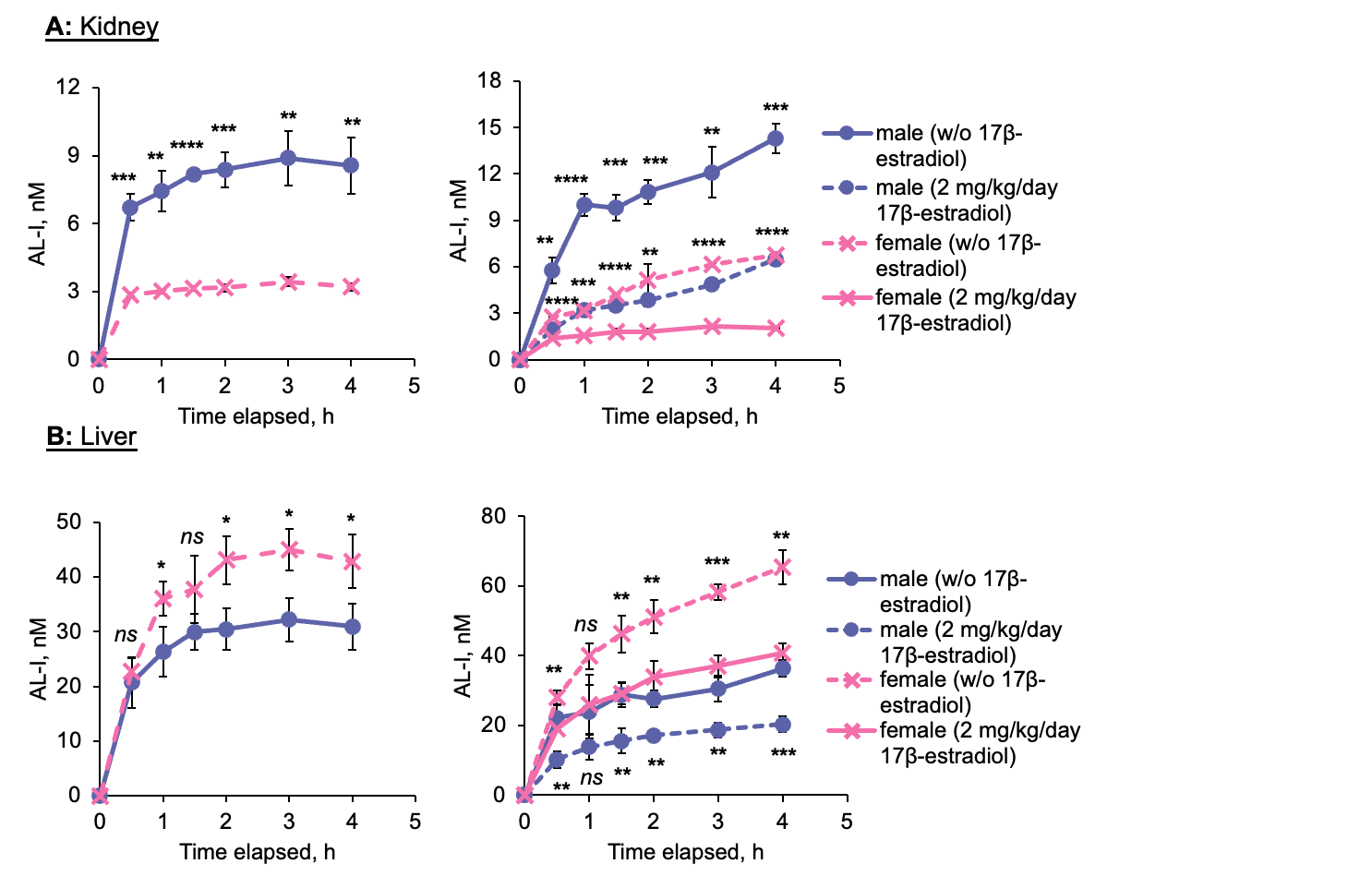
**

**Fig. S1.** Sex hormones affects the metabolic activation of aristolochic acid I, thus the concentrations of aristolactam I in kidneys and livers homogenates prepared from male and female mice. Time profiles of aristolactam I formation by incubation of AA-I with (A) kidneys and (B) livers homogenates prepared from male (*n =* 3) and female (*n =* 3) mice without any treatment and treated with 17β-estradiol for one week. The data represent mean ± SD for three independent experiments. 2-tailed unpaired Student’s *t*-test at 95% confidence interval was conducted to compare the level of aristolactam I in kidney and liver homogenate between male and female mice or between control and 17β-estradiol treatment groups at the same time point. *ns* *p* > 0.05; *, *p* < 0.05; **, *p* < 0.01; ***, *p* < 0.001; ****, *p* < 0.0001.

**
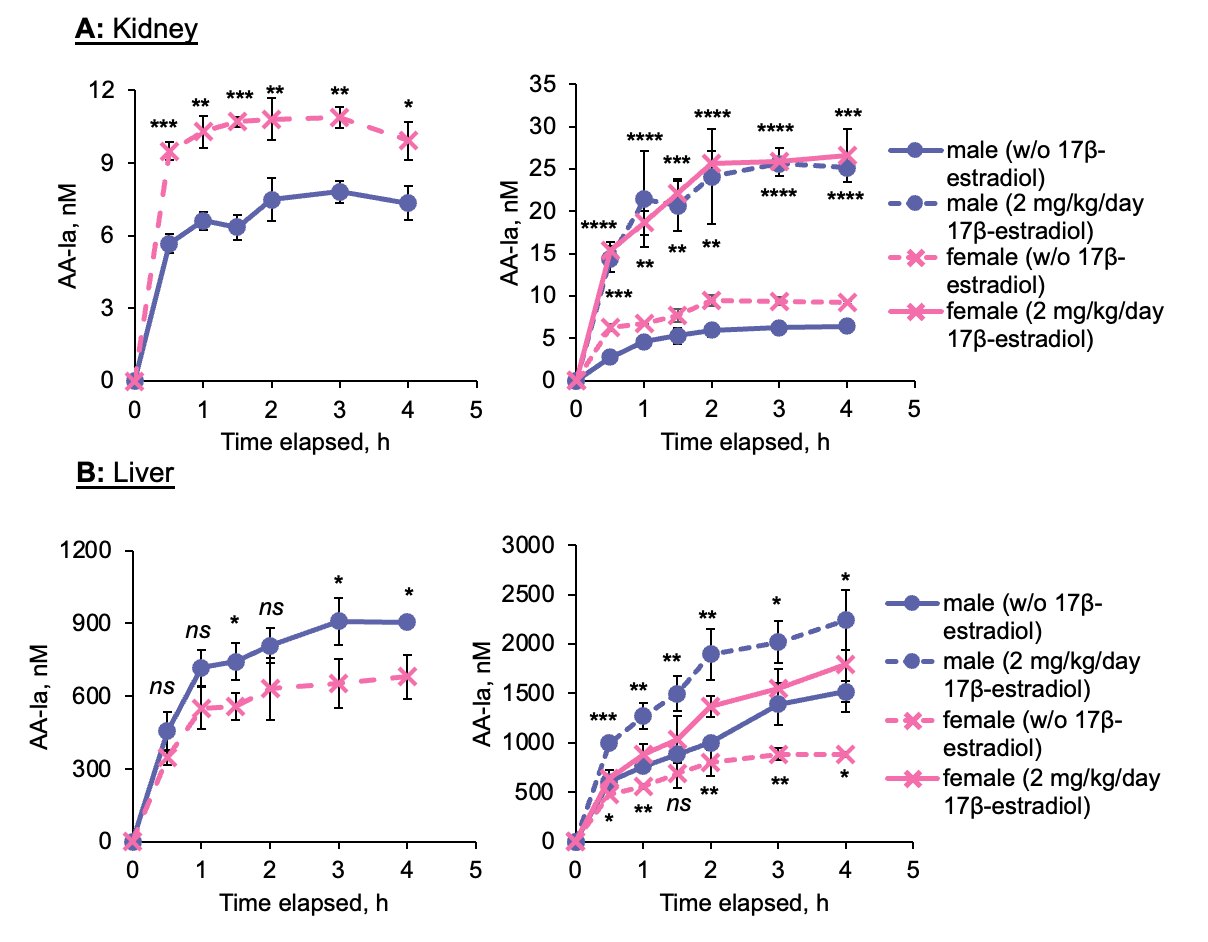
**

**Fig. S2.** Sex hormones affects the metabolic deactivation of aristolochic acid I, thus the concentrations of aristolochic acid Ia in kidneys and livers homogenates prepared from male and female mice. Time profiles of aristolochic acid Ia formation by incubation of AA-I with (A) kidneys and (B) livers homogenates prepared from male (*n =* 3) and female (*n =* 3) mice without any treatment and treated with 17β-estradiol for one week. The data represent mean ± SD for three independent experiments. 2-tailed unpaired Student’s *t*-test at 95% confidence interval was conducted to compare the level of aristolochic acid Ia in kidney and liver homogenate between male and female mice or between control and 17β-estradiol treatment groups at the same time point. *ns* *p* > 0.05; *, *p* < 0.05; **, *p* < 0.01; ***, *p* < 0.001; ****, *p* < 0.0001.

**Table S1.** LC gradient, MS source parameters, and MS compound parameters for LC-MS/MS analysis of (A) ALI-dA adducts, (B) AA-I and AL-I.

1. **ALI-dA adduct analysis**

*LC gradient*

| Time, min | Flow rate, mL/min | % A | % B |
| --- | --- | --- | --- |
| 0.00 | 0.35 | 98.0 | 2.0 |
| 2.00 | 0.35 | 98.0 | 2.0 |
| 3.00 | 0.35 | 70.0 | 30.0 |
| 6.00 | 0.35 | 30.0 | 70.0 |
| 7.00 | 0.35 | 0.0 | 100.0 |
| 11.00 | 0.35 | 0.0 | 100.0 |
| 11.10 | 0.35 | 98.0 | 2.0 |
| 13.50 | 0.35 | 98.0 | 2.0 |

A: 0.1% acetic acid in water; B: acetonitrile

*ESI source parameter*

| Capillary Voltage, kV | 2 |
| --- | --- |
| Source Temperature, ^o^C | 150 |
| Desolvation Temperature, ^o^C | 500 |
| Cone gas flow, L/h | 300 |
| Desolvation gas flow, L/h | 1000 |

*MS compound parameter*

|  | Parent ion,  *m/z* | Daughter ion,  *m/z* | Cone voltage,  V | Collision energy,  eV |
| --- | --- | --- | --- | --- |
| ALI-dA | 543 | 427 | 40 | 20 |
|  | 543 | 292 | 40 | 40 |
| ^15^N_5_-ALI-dA | 548 | 432 | 40 | 20 |

1. **AL-I and AA-Ia analysis**

*LC gradient*

| Time, min | Flow rate, mL/min | % A | % B |
| --- | --- | --- | --- |
| 0.00 | 0.40 | 99.0 | 1.0 |
| 2.00 | 0.40 | 99.0 | 1.0 |
| 8.00 | 0.40 | 0.0 | 100.0 |
| 10.00 | 0.40 | 0.0 | 100.0 |
| 10.10 | 0.40 | 99.0 | 1.0 |
| 12.00 | 0.40 | 99.0 | 1.0 |

A: 0.2% acetic acid and 0.01M ammonium acetate in water; B: acetonitrile

*ESI source parameter*

| Curtain Gas, psi | 25 |
| --- | --- |
| Collision Gas | High |
| IonSpray Voltage, V | 5500 |
| Temperature, °C | 500 |
| Ion Source Gas 1, psi | 30 |
| Ion Source Gas 2, psi | 60 |

*MS compound parameter*

|  | Parent ion,  *m/z* | Daughter ion,  *m/z* | ^a^DP, V | ^b^EP, V | ^c^CE, V | ^d^CXP, V |
| --- | --- | --- | --- | --- | --- | --- |
| AL-I | 294 | 279 | 80 | 14 | 40 | 6 |
|  | 294 | 251 | 80 | 14 | 50 | 6 |
| AA-Ia | 345 | 310 | 40 | 9.5 | 20 | 20 |
|  | 345 | 284 | 40 | 9.5 | 25 | 30 |
| benz[*cd*]indol-2(1*H*)-one | 170 | 115 | 110 | 5 | 50 | 11 |

^a^DP: Declustering Potential

^b^EP: Entrance Potential

^c^CE: Collision Energy

^d^CXP: Collision Cell Exit Potential

**Reference**

1. Tang L, Zhou J, Yang CH, Xia BJ, Hu M, Liu ZQ (2012) Systematic studies of sulfation and glucuronidation of 12 flavonoids in the mouse liver S9 fraction reveal both unique and shared positional preferences. J. Agric. Food Chem. 60: 3223–3233. <https://doi.org/10.1021/jf201987k>
